# ADHD classification by a texture analysis of anatomical brain MRI data

**DOI:** 10.3389/fnsys.2012.00066

**Published:** 2012-09-18

**Authors:** Che-Wei Chang, Chien-Chang Ho, Jyh-Horng Chen

**Affiliations:** ^1^Interdisciplinary MRI/MRS Lab, National Taiwan UniversityTaipei, Taiwan; ^2^Department of Electrical Engineering, National Taiwan UniversityTaipei, Taiwan

**Keywords:** ADHD, ADHD200 global competition, brain anatomical MRI, isotropic local binary patterns on three orthogona, LBP, local binary patterns, MRI, texture analysis

## Abstract

The ADHD-200 Global Competition provides an excellent opportunity for building diagnostic classifiers of Attention-Deficit/Hyperactivity Disorder (ADHD) based on resting-state functional MRI (rs-fMRI) and structural MRI data. Here, we introduce a simple method to classify ADHD based on morphological information without using functional data. Our test results show that the accuracy of this approach is competitive with methods based on rs-fMRI data. We used isotropic local binary patterns on three orthogonal planes (LBP-TOP) to extract features from MR brain images. Subsequently, support vector machines (SVM) were used to develop classification models based on the extracted features. In this study, a total of 436 male subjects (210 with ADHD and 226 controls) were analyzed to show the discriminative power of the method. To analyze the properties of this approach, we tested disparate LBP-TOP features from various parcellations and different image resolutions. Additionally, morphological information using a single brain tissue type (i.e., gray matter (GM), white matter (WM), and CSF) was tested. The highest accuracy we achieved was 0.6995. The LBP-TOP was found to provide better discriminative power using whole-brain data as the input. Datasets with higher resolution can train models with increased accuracy. The information from GM plays a more important role than that of other tissue types. These results and the properties of LBP-TOP suggest that most of the disparate feature distribution comes from different patterns of cortical folding. Using LBP-TOP, we provide an ADHD classification model based only on anatomical information, which is easier to obtain in the clinical environment and which is simpler to preprocess compared with rs-fMRI data.

## Introduction

Attention-Deficit/Hyperactivity Disorder (ADHD) is a multifactorial and clinically heterogeneous disorder, which is highly prevalent in children worldwide. It is estimated that 5–10% of school-age children and 4% of adults suffer from ADHD (Biederman, 2005). The negative impact of ADHD on patients, their families, and society make ADHD a major public health problem (Ferguson, 2000). However, an objective biological tool to diagnose ADHD is still unavailable. Foreseeing the importance, the organizers of the ADHD-200 Global Competition have collected functional and anatomical ADHD MRI datasets of an unprecedented scale, which are accessible via the Internet (http://fcon_1000.projects.nitrc.org/indi/adhd200/). This work provides an important opportunity for researchers all over the world to study brain changes in ADHD subjects based on numerous brain MRI images.

During the ADHD-200 global competition, we tried many strategies to discriminate ADHD patients from control subjects based on resting-state fMRI (rs-fMRI) and anatomical brain image data. We found that the brain morphological changes described by a 3D texture analysis can be used to distinguish children with ADHD from typically developing children (TDC). These structural image-based models demonstrated similar accuracy compared with our models based on rs-fMRI data. In the present study, we describe and analyze the 3D texture analysis method.

It is not easy to construct a classification rule to distinguish ADHD from TDC subjects. ADHD is a complex disorder with a composite etiology (Faraone and Mick, 2010). No simple existing indicators can be used to diagnose ADHD at present. Currently, the Diagnostic and Statistical Manual of Mental Disorders, 4th ed., text revision (DSM-IV-TR) is most often used for diagnostic criteria for ADHD. Some ADHD criteria are based on subjective descriptions by a child's parents or teachers and not on objective analysis tools. Recent research has demonstrated that using different versions of the DSM or disparate sources of collateral information can significantly affect the calculated prevalence of ADHD (Polanczyk et al., 2007). Moreover, both sex and age play important roles in the development of ADHD. These factors also increase the complexity of building a diagnostic tool (Biederman, 2005). All the aforementioned factors make it challenging to build an efficient classification model for ADHD.

Additionally, approaches based on rs-fMRI data suffer from unstable echo planar imaging (EPI) and involve sophisticated data preprocessing steps. For these reasons, building a classification model based on rs-fMRI data from multiple research sites involves difficult manipulations of large data sets and is not efficient.

However, structural brain images are of high quality and are more stable with better resolution compared with rs-fMRI data. We hypothesized that structural brain images might contain more information from which to build a discriminative model. Although ADHD is not believed to result from morphological changes in the brain, several studies have shown that anatomical differences associated with ADHD can be found in MR images (Qiu et al., 2011). Large changes in volume and structural differences in the cerebral cortex have also been reported using MRI methodologies, such as anatomical MRI and diffusion tensor imaging (Kobel et al., 2010). Hence, we set forth to develop an ADHD classification method based on morphological changes. Notably, after using 3D texture descriptors to extract features from brain anatomical data, we found that morphological changes provided information that could discriminate ADHD from TDC subjects.

In this paper, to describe brain morphology, we introduce a feature extraction method based on texture point of view using the isotropic local binary patterns on three orthogonal planes (LBP-TOP). After extracting features using LBP-TOP, we trained a support vector machines (SVM) model and built an ADHD classification model based on the extracted features.

Texture analysis based on local binary patterns (LBP) has recently been shown to have excellent discriminative power for many applications in the domain of computer vision (Ojala et al., 2002; Inen et al., 2011). LBP was originally designed to extract features from various textured images, such as organic fibers, wood, and fabric (Ojala et al., 1996). After decades of development, it was also found to be useful for extracting the features from other types of images, such as face description (Ahonen et al., 2006), image segmentation, and other applications (Inen et al., 2011). Furthermore, it can be used as a spatiotemporal descriptor for motion and activity analysis (Zhao and Pietikainen, 2007). In the domain of computer vision, LBP is an efficient and robust method for extracting information from morphology (Inen et al., 2011).

Recently, research has been directed toward using LBP to extract features of medical images (Unay et al., 2007). Research has been performed on finger vein recognition (Rosdi et al., 2011), image annotation (Tommasi and Orabona, 2010) and medical image retrieval (Qian et al., 2011). Most of the research with medical images has used 2D-LBP approaches to extract features. Few studies have used 3D-LBP approaches to describe the features of medical images.

In the present study, we build an ADHD classification model using LBP-TOP features and SVM. Different registration methods, LBP-TOP settings, and source brain image resolutions were utilized to test the properties of this method. A simple and efficient feature selection method was introduced to create a more robust model. We built classification models based on three basic brain tissues: gray matter (GM), white matter (WM), and CSF. Our results demonstrate that it is possible to build an ADHD classification model based on LBP-TOP features. We found that GM data provide the most salient information for discriminating ADHD from TDC subjects.

## LBP-TOP

**Figure 1:**
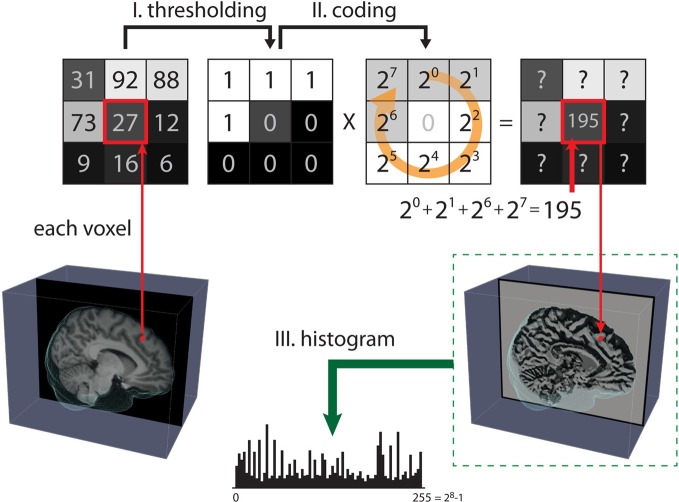
**Steps for computing the local binary pattern (LBP) operator**. The LBP of 2D images aims to map all pixels from the grayscale pattern space onto the binary pattern space in three steps. **Step (I)**: A small window is defined by a radius R and the total number of neighborhood pixels *P* (in this case, *p* = 8). **Step (II)**: The neighborhood of each pixel is thresholded by the value of the center pixel, resulting in a binary number. Then, the resulting code of the center pixel is given as a weighted sum of the binary number of its neighbors. **Step (III)**: After all the LBP codes of an image are computed, the texture features are defined by building a histogram over specific regions or over the whole image.

**Figure 2:**
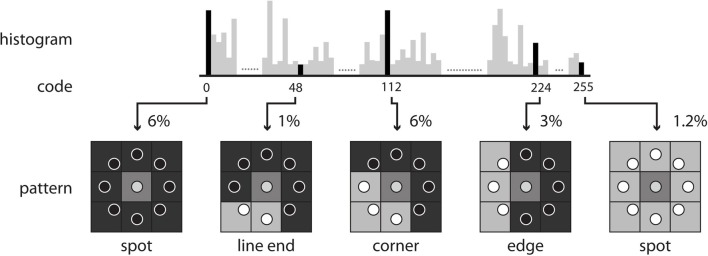
**Some examples of texture patterns encoded by LBP**.

LBP is a simple and efficient image texture operator introduced by Ojala et al. (1996, 2002). Figure 1 shows the three steps for computing LBP on 2D images. The LBP_*P*,*R*_ operator can be defined as

(1) $${\text{LBP}}_{P,R}=\sum_{p\;=0}^{P\;-1}\text{sign}(v_{p}-v_{c})2^{P}$$

$$\text{sign}(x)=\{\begin{array}{c} 1,\;x\geq 0\\ 0,\;x<0 \end{array}$$

where *v*_*c*_ and *v*_*p*_ are the values of the center pixel and neighborhood pixels with radius *R*, respectively, *P* is the total number of neighborhood pixels, and *R* is the radius in pixel. After the LBP codes for all voxels in an image are computed, the histogram of the codes computed over specific regions or over the whole image can be used as a texture descriptor. Therefore, each bin of the histogram can be regarded as a “micro-texton” encoded by LBP (Hadid et al., 2004). Figure 2 demonstrates patterns encoded by these histogram bins. Any morphological changes would modify the distribution of the codes, resulting in alterations to the histogram. Therefore, the histogram of the computed LBP codes is a good descriptor for comparing changes between images.

**Figure 3:**
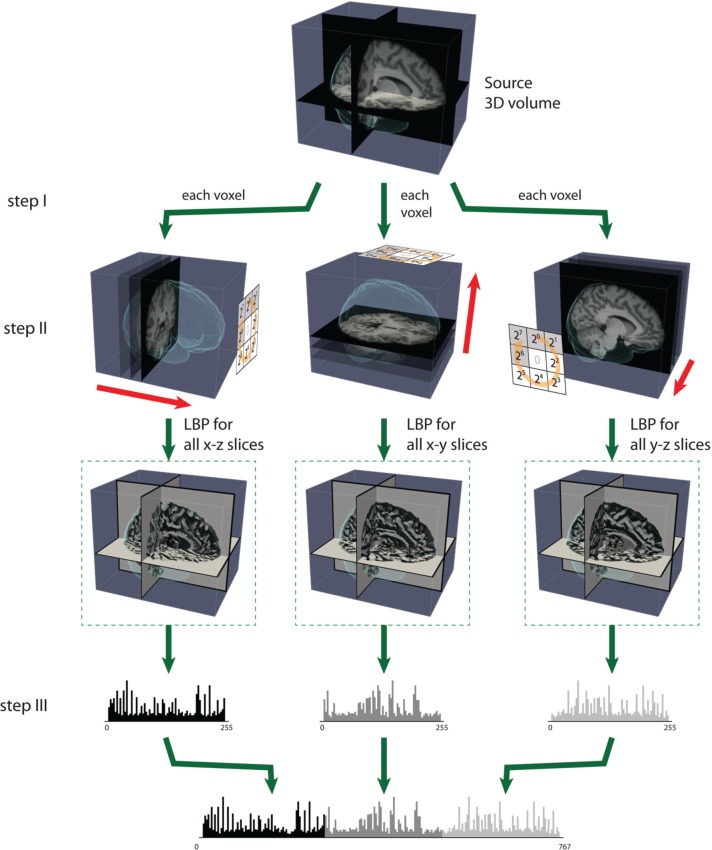
**The steps of computing LBP on three orthogonal planes (LBP-TOP)**. **Step (I)**: A small window is defined by a radius *R* and the total number of neighborhood pixels *P*. **Step (II)**: LBP codes are computed for three orthogonal directions (x, y, z). Each voxel is encoded based on the three orthogonal planes (xy, yz, and xz). After the LBP of each direction is computed, the texture features can be computed by building the histogram over a specific volume or over the whole-brain volume. **Step (III)**: The histograms from the three planes are combined to build the resulting histogram, which represents the texture features of the selected volume.

For 3D data, Zhao et al. have proposed simplifying spatiotemporal descriptors by concatenating LBP on three orthogonal planes (LBP-TOP), i.e., the xy, xt, and yt planes (Zhao and Pietikainen, 2007). Here, we used LBP-TOP to describe brain volume data. Therefore, we replaced the *t* dimension with the *z* dimension. We propose using the same radius for *x*, *y*, and *z* for LBP-TOP. Figure 3 illustrates the specific steps for computing LBP-TOP on 3D-volume data.

**Figure 4:**
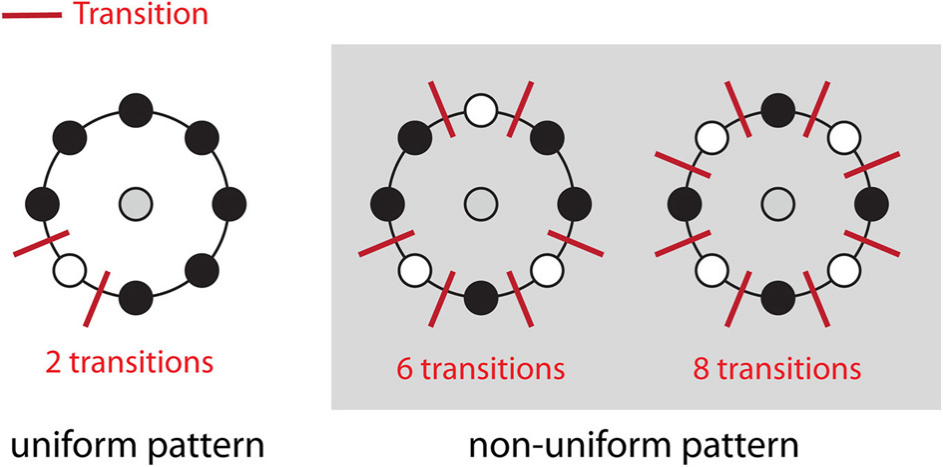
**Uniform patterns**. Using circular neighborhoods, *U* is calculated as the number of bitwise transitions from 0 to 1 or vice versa. An LBP pattern is considered uniform if *U* is less than or equal to 2. Here, we show three simple examples of uniform and non-uniform patterns. A list of all uniform LBP patterns with eight neighbors has previously been published (Inen et al., 2011).

The direct output of LBP-TOP is an over-complete set of features. One method for selecting the most informative bins from the raw histogram is to map “uniform patterns.” Figure 4 shows the rule of uniform patterns and some examples. Ojala et al. observed that in image classification problems, such as face and texture classification, the major patterns are uniform patterns. Non-uniform patterns rarely exist (Ojala et al., 2002). In uniform LBP mapping, there is a separate output label for each uniform pattern, and all non-uniform patterns are assigned to a single label. In the case of LBP with eight neighbors, after mapping uniform patterns, the length of the histogram bins for whole-brain images was reduced from 255 to 259.

## Materials and methods

### Participants

To best demonstrate the discriminative power of LBP-TOP, only male subjects were used to control for the known sex-based differences in ADHD subjects (Biederman, 2005; Polanczyk et al., 2007). Male data from the Kennedy Krieger Institute (KKI), the NeuroIMAGE sample (NeuroIMAGE), the New York University Child Study Center (NYU), Oregon Health and Science University (OHSU), Peking University (Peking_1, Peking_2, and Peking_3), and the University of Pittsburgh (Pittsburgh) were selected for analysis in this study. We ruled out using the dataset from Washington University because it was not in the test set of the ADHD-200 global competition and no ADHD subject in it. Five subjects (0010016, 0010027, 0010055, 0010098, and 0010127) in the NYU dataset were excluded because no anatomical data existed for them. Subject 0010013 in the NYU dataset was also excluded because some of the brain in the anatomical image was cropped during the face removal process. ADHD hyperactive-type subjects were excluded due to the small number of such subjects in the dataset.

Therefore, the ADHD subjects in this study were of both the ADHD combined type and the ADHD inattentive type. A total of 436 male subjects (210 ADHD subjects and 226 TDC, mean age = 12.12 ± 2.95) were used in this study. The distributions of subjects by age and by type of ADHD are shown in **Tables S1** and **S2**. A list of all subjects can be found in **Table S3**. The detailed phenotype of each subject can be found on the website for the ADHD-200 global competition (http://fcon_1000.projects.nitrc.org/indi/adhd200/).

### Diagnostics of ADHD

Table 1 shows a brief summary of the diagnosis criteria used by each site. The sites used different ADHD criteria, intellectual evaluations, and sources of collateral information.

**Table 1:** **A brief summary of the different diagnostic criteria used by each site**.

**Site**	**ADHD criteria**	**Intelligence evaluation**	**Source of information**
Kennedy Krieger Institute (KKI)	DICA-IV	WISC-IV	Parents
	DuPaul		Subjects
	CPRS-R		
	DSM-IV		
NeuroIMAGE sample (NeuroIMAGE)	KSADS-PL	WASI	Parents
	CPRS-LV		Subjects
New York University Child Study Center (NYU)	KSADS-PL	WASI	Parents
	CPRS-LV		Subjects
Oregon Health and Science University (OHSU)	KSADS-I	WISC-IV	Parents
	CPTRS-III		Teachers
			Subjects
Peking University (Peking)	C-DIS-IV	WISCC-R	Parents
	KSADS-PL		
University of Pittsburgh (Pittsburgh)	N/A	WASI	N/A

Abbreviations are as follows: **C-DIS-IV**, Computerized Diagnostic Interview Schedule IV; **CPRS-LV**, Conners' Parent Rating Scale-Revised, Long version; **CPRS-R**, Conners' Parent Rating Scale-Revised, Long Form; **CPTRS-III**, parent and teacher Connors' Rating Scale, Third Edition; **DuPaul**, DuPaul ADHD Rating Scale IV; **DSM-IV**, Diagnostic and Statistical Manual of Mental Disorders, Fourth Edition; **KSADS-I**, Kiddie Schedule for Affective Disorders and Schizophrenia; **KSADS-PL**, Schedule of Affective Disorders and Schizophrenia for Children—Present and Lifetime Version; **WASI**, Wechsler Abbreviated Scale of Intelligence; **WISCC-R**, Intelligence Scale for Chinese Children-Revised; and **WISC-IV**, Wechsler Intelligence Scale for Children, Fourth Edition. Details can be found on the ADHD-200 global competition website (http://fcon_1000.projects.nitrc.org/indi/adhd200/).

### Data preprocessing

**Figure 5:**
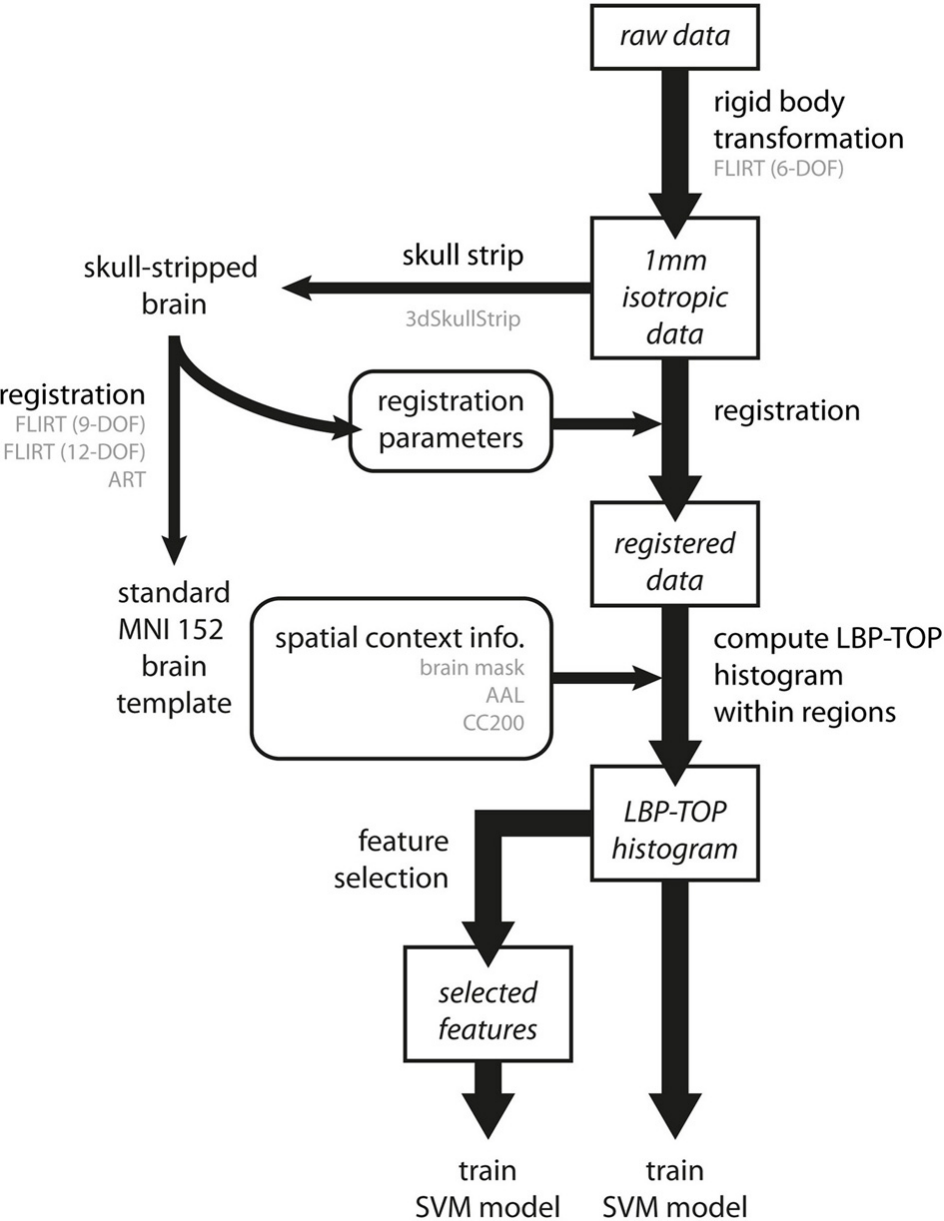
**Overview of the data analysis procedure**. Preprocessing involves three steps. First, all raw data are transformed into a 1 mm isotropic volume using the rigid body transformation as performed by FLIRT with 6 degrees of freedom (6-DOF). Second, brain images are registered to standard MNI152 space by linear (FLIRT with 9-DOF and FLIRT with 12-DOF) and non-linear (ART) registration methods. To achieve better registration results, the registration parameters were obtained by transforming the skull-stripped brains to the standard MNI152 brain template. Third, we computed the LBP-TOP histograms based on the registered images with various spatial context information (i.e., brain mask, AAL, and CC200). Following these steps, classification models can be trained by directly using the resulting histogram or by using a subset of data after applying the feature selection algorithm. (The major process flow is denoted by the thick line. The minor process flow is denoted by the thin line. The square boxes are the major steps showing how to extract features from raw data. The round boxes are the parameters or information needed for the process flow. Texts in gray color indicate the methods or the subtypes the process step used.)

An overview of the data analysis procedure is shown in Figure 5.

The details of each step are described below.

#### Registration methods

Linear registrations with 9 degree of freedom (9-DOF) and 12-DOF were performed using the linear multimodality registration method developed by Oxford FSL FLIRT (Jenkinson et al., 2002; Smith et al., 2004). All images were transformed to standard MNI152 space by FLIRT with 6-DOF (rigid-body transformation). The results of FLIRT with 6-DOF were then linearly transformed by FLIRT with 9-DOF (rigid-body + independent scaling). The results of FLIRT with 9-DOF were also linearly transformed by FLIRT with 12-DOF (rigid-body + scales + skews). Non-linear normalization procedures were performed using the automated registration tool (ART). ART was developed by Ardekani et al. (2005) and can be downloaded from http://www.nitrc.org/projects/art/. Klein and colleagues demonstrated that ART provides better efficiency and consistency than other non-linear registration methods (Klein et al., 2009).

#### 3D skull striping

To obtain better registration results, skull stripping was performed prior to using the registration algorithm, using the 3DSkullStrip algorithm (Smith, 2002) developed by AFNI (Cox, 1996). 3DSkullStrip has proven to be a relatively robust skull-stripping algorithm (Iglesias et al., 2011). However, it is not a perfect tool. Incomplete skull stripping can result in a loss of information from some brain regions. Hence, we applied the transformation parameters for skull-stripped brains to the original whole-brain images to create the input for the LBP-TOP algorithm.

#### Spatial context information and brain parcellations

To examine different spatial context information, we performed three separate parcellations in this study.

First, a simple brain mask in MNI152 space, as provided by FSL, was used to compute the total histogram of the whole-brain volume. Second, to introduce spatial context based on brain anatomical information, the widely used automated anatomical labeling (AAL) template with 116 regions was used (Tzourio-Mazoyer et al., 2002). Finally, we also used an atlas derived from functionally parcellating the resting state data (Craddock et al., 2012). A 200 ROI version with 190 regions of spatially constrained parcellation (CC200) was used to introduce the spatial context information based on rs-fMRI data. The CC200 functional parcellation template made for the competition was kindly provided by Cameron Craddock. Details of the construction of CC200 have been previously published (Craddock et al., 2012) and can also be found on the Athena preprocessing strategies page of the ADHD-200 preprocessed data website: http://www.nitrc.org/plugins/mwiki/index.php/neurobureau:AthenaPipeline.

### Computation of LBP-TOP

The LBP-TOP algorithm was implemented using Java to build the LBP-TOP map from the structural image. All resulting LBP-TOP histograms were mapped for the detection of uniform patterns. Preliminary testing (not shown) demonstrated that only the LBP-TOP with eight neighbors provided sufficient information to classify ADHD within a reasonable processing time. Therefore, only tests with eight neighbors are shown here.

### Classifiers

A *k* nearest neighbor classifier (KNN, *K* = 1) was used to show the baseline of the discriminative power of LBP-TOP. Moreover, an efficient and widely used classifier, SVM, was used in this work (Boser et al., 1992). SVM maps training data into high-dimensional feature space to find the separating hyperplane with the maximal margin. Due to the large feature size of LBP-TOP results, we used linear SVM for greater efficiency. LIBLINEAR (Fan et al., 2008) was chosen for use because of its optimization for linear SVM.

### Feature selection

After introducing spatial context information, the LBP-TOP histogram bins become an over-completed feature set. To build a more efficient and robust classification model, a feature selection method is needed. Moreover, by only selecting the most important features, we can combine features from various points of view. For example, we can combine features from different LBP-TOP results based on dissimilar radii.

Feature selection based on the linear SVM has proven to be efficient and useful for gene selection, document classification, and many other applications (Brank et al., 2002; Chang and Lin, 2008).

For any test subject *x*, the decision function of linear SVM is

(2) $$P(x)=\text{sign}(w^{T}x+b)$$

where **x** is the feature vector, *b* is a constant, and **w** is the weight vector. Each value of **w** denotes the weight of each feature. The larger the absolute value of *w*_*j*_, the more important the *j*^th^ feature is in deciding the result.

After training a linear SVM model, the **w** in (2) can be used as a relative importance index. Therefore, we can build a simpler model using the top *n* important features.

For combining features from different point of views, we first trained a linear SVM model using feature groups and ranked features by the absolute weights of the model. Only half of the features remained. Then, we combine these features with the features from a second feature group and trained another linear SVM model. Similarly, only half of the features were chosen to be merged into the next feature group. Using this iterative procedure, we combined various feature groups and found the most important features among these feature groups. **Algorithm 1** shows steps of this iteration. Given a set of *N* subjects and *K* different feature groups, for each training dataset of our 10-fold cross-validation, we use **Algorithm 1** to select and combine the most important features.

Algorithm 1 **The algorithm of feature selection.**

**Input:** Training dataset $D={\left\{{\left\{x_{k,n},\;y_{n}\right\}}_{n\;=1}^{N}\right\}}_{k=1}^{K}$, **x**_*k*_ is different feature set based on various setting

**Output:** Training dataset with selected features *S* = {**s**_*n*_, *y*_*n*_}^N^_*n* = 1_

For *k* = 1,…, *K*

1. For each subject *n*, add all features of **x**_*k*, *n*_ to **s**_*n*_.
2. Use grid search with 10-fold cross-validation to find the best penalty parameter of linear-SVM based on *S*.
3. Train a linear-SVM model based on *S* using the best penalty parameter.
4. Sort the features of **s**_*n*_ based on the absolute weights of the linear-SVM model.
^*^5. For each subject *n*, drop the last half features of **s**_*n*_.

Loop

^*^Due to the small number of features revealed when analyzing the whole-brain region, we simply combined all the features and do not drop the last half of them.

### Brain segmentation

**Figure 6:**
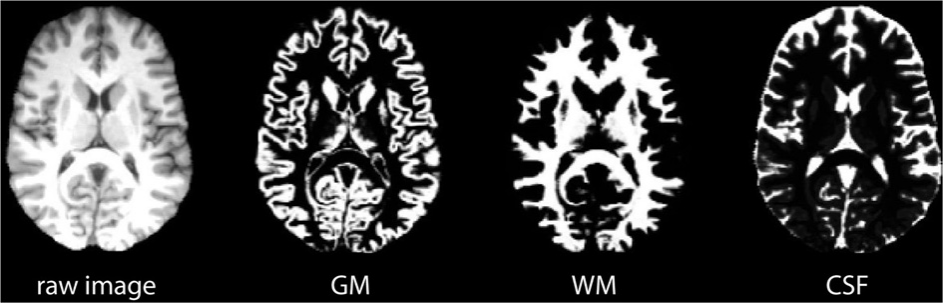
**Examples of brain probability maps based on gray matter (GM), white matter (WM), and CSF**.

FSL's automated segmentation toolbox (FAST) was used to segment raw brain images into GM, WM and CSF (Zhang et al., 2001; Smith et al., 2004). Figure 6 shows an example of a resulting probability map. The three tissue probability maps were analyzed following the same procedure described in Figure 5.

### Reference models based on rs-fMRI features

To compare the results of discriminative models based on rs-fMRI data, we used a simple and easily repeatable approach. Briefly, for the preprocessing of rs-fMRI data, we used the extracted timecourses from the Athena preprocessed data, which can be download from the ADHD-200 Preprocessed Data website. Details of the specific preprocessing steps can be found on the website.

The timecourses of the AAL and CC200 parcellations used in LBP-TOP study were chosen for comparison. The extracted timecourses files, ADHD200_AAL_TCs_filtfix.tar.gz and ADHD200_CC200_TCs_filtfix.tar.gz, can be found on the ADHD-200 Preprocessed Data website. The correlation coefficients between each pair of regions were computed based on their extracted timecourses. For example, there are 116 regions in the AAL parcellation. Therefore, 6670 correlation coefficients can be computed based on the 6670 ROI pairs. All the correlation coefficients were used as features for the linear SVM ADHD classifier. The results of each model were validated using the same cross-validation settings used in the LBP-TOP studies. As described on the ADHD-200 Preprocessed Data website, the nuisance variance for the extracted time series of each region was removed, with or without use of a band-pass filter (0.009 Hz–0.08 Hz), and blurred with a 6 mm FWHM Gaussian filter. Both time series, with or without filtering by a band-pass filter, were tested.

### Evaluation

All tests in this study were evaluated by 10-fold cross-validation. We randomly partitioned the 436 subjects into 10 subgroups. For each step of cross-validation, one subgroup was used as a test data set, and the remaining nine subgroups were pooled as a training data set. After 10 cross-validations, the test results of all 10 subgroups were combined to build the accuracy of the estimation of each model. To facilitate comparison of the results, the same 10-fold cross-validation set was used in all evaluations.

We used grid searching to find the best penalty parameter C for linear SVM for each training dataset. That is, another 10-fold cross-validation was applied to each training dataset with several candidate values of *C*, and we chose the parameter *C* that led to the highest accuracy.

While performing feature selection, the assignment of optimal feature weights can be achieved when the optimal value of *C* is chosen during each round of cross-validation. After that, we evaluated the effect of feature number using each testing dataset. Then, we combined the results of 10 test dataset to build the accuracy of different feature numbers.

### Statistical tests

To show the classifier has learned a structure in the data, we compute the *p*-value against the null distribution using permutation tests (Good, 2000; Ojala and Garriga, 2010). The null hypothesis of permutation test is that the labels are independent of the features. Therefore, one can learn almost same accuracy using random labeled data set. By randomly permuting the labels of the data set, permutation tests can measure how likely the observed accuracy is learned by chance. The permutation-based *p*-value is defined by

$$p=\frac{\left|\left\{D^{\prime }\in \overset{⌢}{D}:e(c,\;D^{\prime })\leq e(c,\;D)\right\}\right|+1}{k+1}$$

where *D* is the original labeled data, *e*(*c*, *D*) denotes the error of classifier *c* learned from *D*, and $\overset{⌢}{D}$ is a set of *k* randomized versions *D*′ of *D* (Ojala and Garriga, 2010). In this work, the *e*(*c*, *D*) was estimated by same 10-fold cross-validation with other tests. One hundred randomized sets of each test were used to estimate the *p*-values (*k* = 100).

To compare different approaches of this work, McNemar's tests were applied to compute *p*-values between two approaches (Everitt, 1992; Dietterich, 1998). While comparing two different approaches, confusion matrices of each approach were estimated by same 10-fold cross-validations. Then we compute the *p*-values of McNemar's tests using R (Team, 2012).

## Results

### LBP-TOP

Table 2 shows the 10-fold cross-validation results for different radii (1 mm, 2 mm, and 3 mm) for the LBP-TOP, various parcellations, linear registrations, and non-linear registrations, respectively. Table 2A shows the baseline accuracy which LBP-TOP can provide with the simple 1 NN classifier. Comparing the results of Tables 2A and 2B, we can find the linear-SVM classifiers can provide better accuracy than 1 NN classifiers. Moreover, some of the properties changed while using different classifiers. The LBP-TOP with a radius equal to 3 mm provided better accuracy than the LBP-TOP for the other two radii in most cases while using linear-SVM classifiers. The same properties cannot be found while using 1 NN approaches. However, there are nonsignificant between different radii in NcNemar's test (**Table S4**). As expected, brain data with ART non-linear registration showed the highest accuracy in almost all cases, especially while using 1 NN as classifiers. Notably, using linearly registered brain data did not greatly reduce accuracy. After apply NcNemar's test, there is no significant difference between registration methods in any cases with linear-SVM classifiers. And only few cases show significant difference between registration methods while using 1 NN approaches (**Table S5**).

**Table 2:** **(A,B) The ADHD-TDC classification accuracy of models based on LBP-TOP features with different registration methods, parcellation, and radius of LBP-TOP, using 1NN and linear-SVM classifiers alternatively. (C,D) The ADHD-TDC classification accuracy of models based on simple rs-fMRI features, using 1NN and linear-SVM classifiers alternatively**.

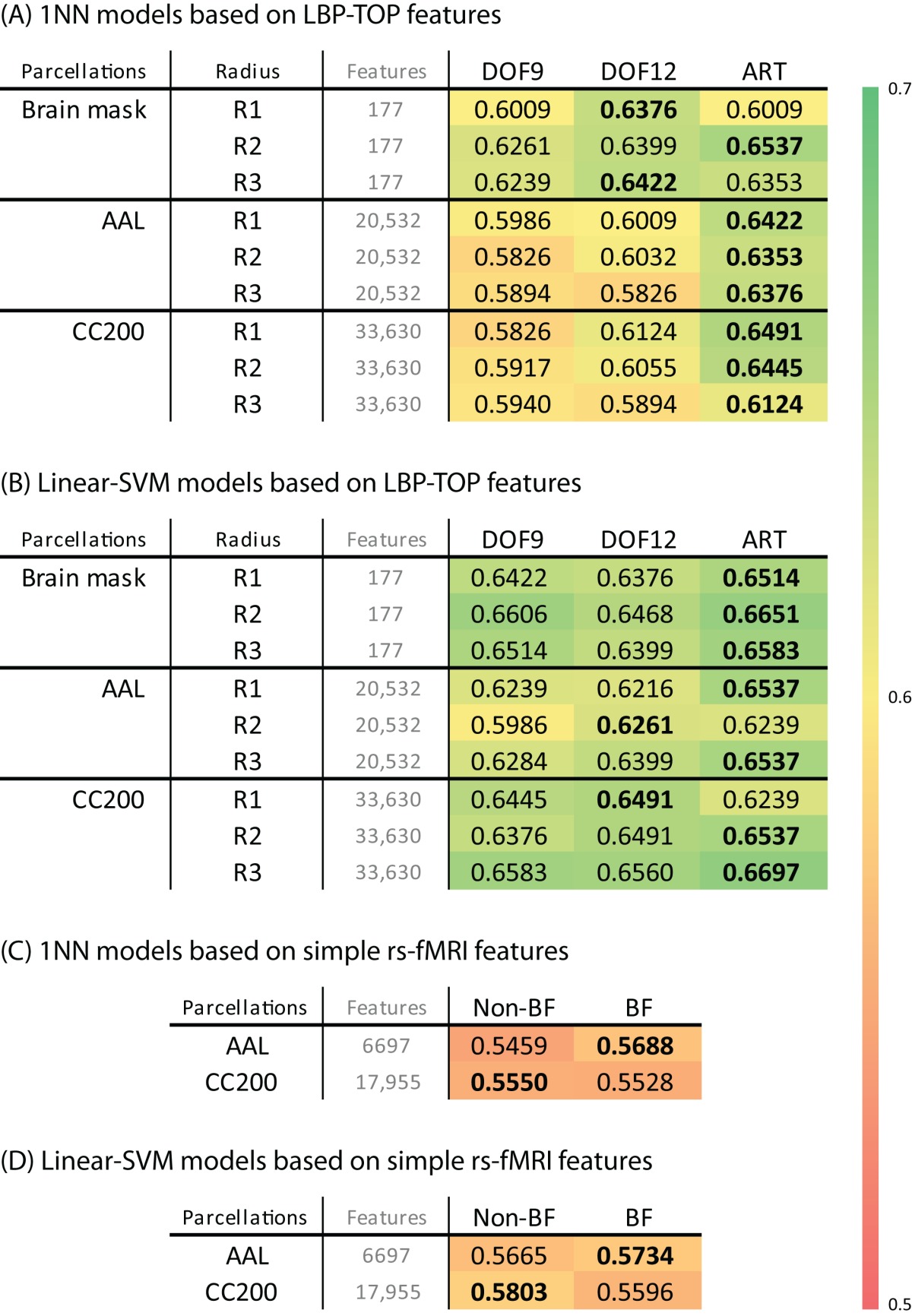

The highest accuracy for each parcellation is denoted by the bold number. Abbreviations are as follows: **R1**, **R2**, and **R3**, LBP-TOP radius in mm; **DOF9**, and **DOF12**, linear registration with 9, and 12 degree of freedom, respectively; **ART**, non-linear registration performed by Automated Registration Tool; **AAL**, automated anatomical labeling template; and **CC200**, spatially constrained parcellation based on rs-fMRI. Abbreviations are as follows: **BF**, rs-fMRI data filtered by a bandpass filter (0.009 Hz ~ 0.08 Hz); and **non-BF**, rs-fMRI data not filtered by a bandpass filter.

Although the resulting feature sizes varied widely (from 177 to 33630 features), accuracy across disparate parcellations was not greatly affected. Models using a histogram computed from the whole-brain region had higher accuracies than models based on other parcellations. Only considering the results of the AAL and CC200 parcellations, the CC200 showed better results most often. This finding may be the result of the greater number of utilized features or the greater number of homogeneous areas in the CC200 parcellation.

The results of reference models based on rs-fMRI features are shown in Table 2. These data indicate that simple approaches to analyzing rs-fMRI data do not discriminate as well as models based on structural information. The McNemar's test between structural features and rs-fMRI features also show significant difference in most cases (Table 3). Based on our experience in the ADHD-200 Global Competition, different preprocessing settings can affect the resulting accuracy. Moreover, combining the results of different rs-fMRI approaches can provide better discriminative power. The results of these simple approaches can be viewed as the baseline of discriminative power that rs-fMRI data can achieve.

**Table 3:** ***p*-values of McNemar's test of linear-SVM models based on rs-fMRI data and LBP-TOP features using non-linear registration**.

**Parcellations**	**Radius**	**Non-BF and LBP-TOP**	**BF and LBP-TOP**	**BF and non-BF**
AAL	R1	**0.0056**	**0.0102**	0.8467
	R2	0.0633	0.1052	
	R3	**0.0043**	**0.0106**	
CC200	R1	0.1586	**0.0454**	0.3619
	R2	**0.0093**	**0.0018**	
	R3	**0.0013**	**0.0002**	

The *p*-values without under lines denote the accuracies of LBP-TOP features are bigger than rs-fMRI data, and the accuracies of rs-fMRI data filtered by a bandpass filter are bigger than rs-fMRI data not filtered. The underlined *p*-values show the inverse relationship. Bold *p*-values correspond to significant results (*p*-value < 0.05). Abbreviations are as follows: **AAL**, automated anatomical labeling template; **CC200**, spatially constrained parcellation based on rs-fMRI; **BF**, rs-fMRI data filtered by a bandpass filter (0.009 Hz–0.08 Hz); and **non-BF**, rs-fMRI data not filtered by a bandpass filter.

### Permutation test of basic models

The results of permutation test in Table 4 shows each approach can learn the class structure in the data. Classifiers based on LBP-TOP features show more significant than approaches based on rs-fMRI data.

**Table 4:** **Permutation test of some results in Table 2**.

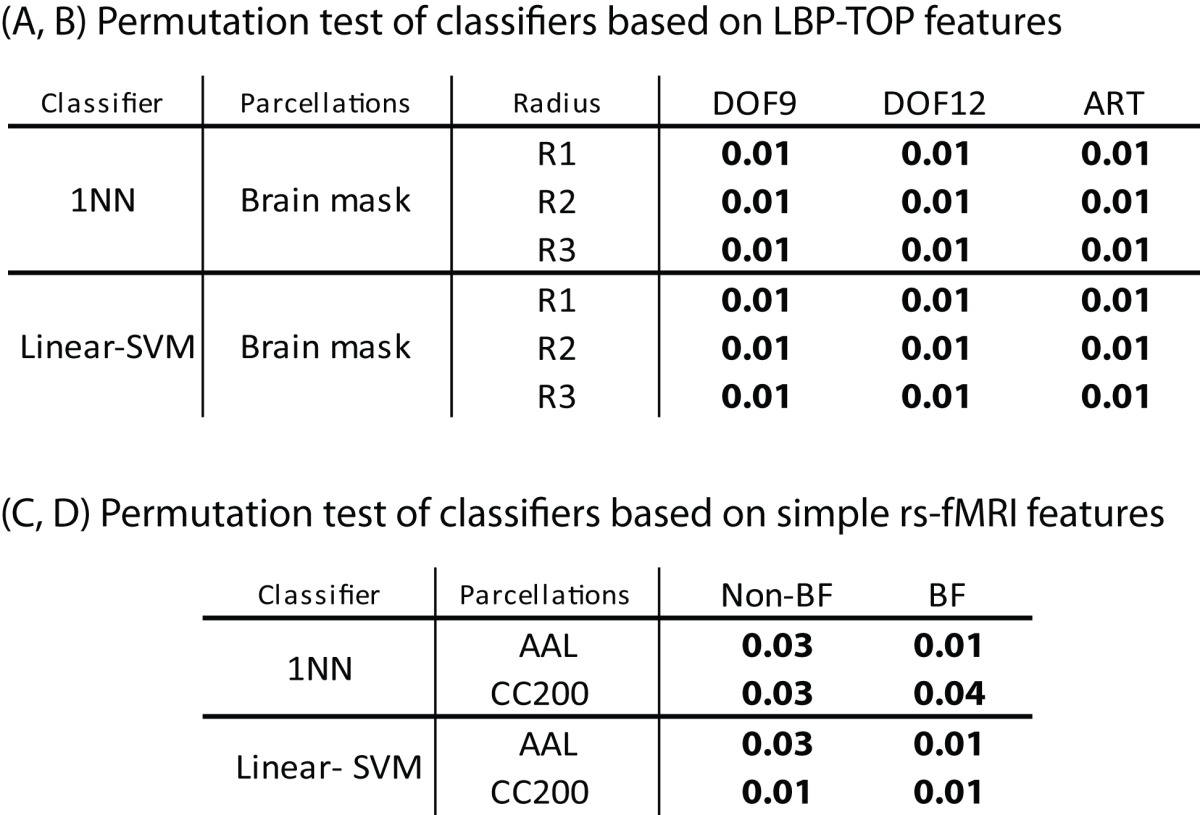

The *p*-values are calculated over 100 randomized sets of each test. The error of each set was estimated by same 10-fold cross-validation of data. Bold *p*-values correspond to significant results (*p*-value < 0.05).

### Feature selection

**Figure 7:**
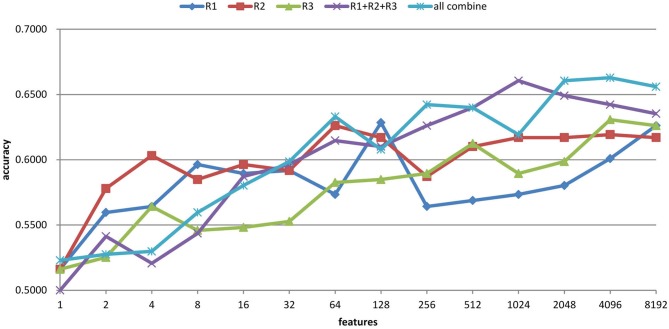
**Feature selection results of ADHD-TDC classification accuracy based on AAL parcellation and the ART non-linear registration method**. R1, R2, and R3 denote the LBP-TOP radii in mm. R1 + R2 + R3 denotes the combination all features from R1, R2, and R3. All combine refers to the combination of all features from different parcellations (i.e., brain mask, AAL, and CC200) and various radii.

The feature selection results with the ART non-linear registration methods are shown in Table 5. When introducing spatial context information (the AAL and CC200 parcellations), only a few features are needed to build a sufficiently accurate classification model. In most cases, using the same number of features but combining features from all the radii of LBP-TOP (R1 + R2 + R3) improves the accuracy of the resulting model. After combining all features based on different parcellations and various radii, we achieved a model with greater accuracy compared with the AAL or CC200 parcellations alone. However, the accuracy of the combined model did not surpass that of the model based on the histogram of the whole-brain region. Figure 7 shows the test results from using different feature groups based on AAL parcellation.

**Table 5:** **ADHD-TDC classification results using LBP-TOP based on the ART non-linear registration method**.

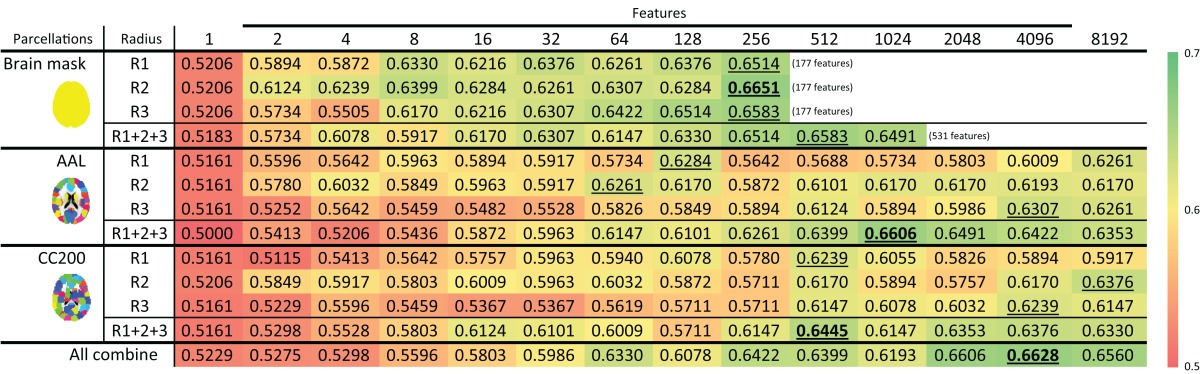

The highest accuracy for each parcellation is denoted by the bold number. The highest accuracy for each row is underlined. **R1**, **R2**, and **R3** denote the LBP-TOP radii in mm. **R1 + R2 + R3** denote the combination of all features from R1, R2, and R3. **All combine** denotes the combination of all features from different parcellations (i.e., brain mask, AAL, and CC200) and various radii. Other abbreviations are as follows: **AAL**, automated anatomical labeling template; and **CC200**, spatially constrained parcellation based on rs-fMRI.

### Resolutions of brain images

Table 6 shows the accuracy of models based on various brain image resolutions. Models utilizing higher resolutions usually had better accuracy. However, models based on the CC200 parcellation had greater accuracy when using 3 × 3 × 3 mm resolution. Nevertheless, higher resolution data generally provided more information for the discrimination of ADHD from TDC subjects.

**Table 6:** **Feature selection results of ADHD-TDC classification accuracy of different resolution of source images**.

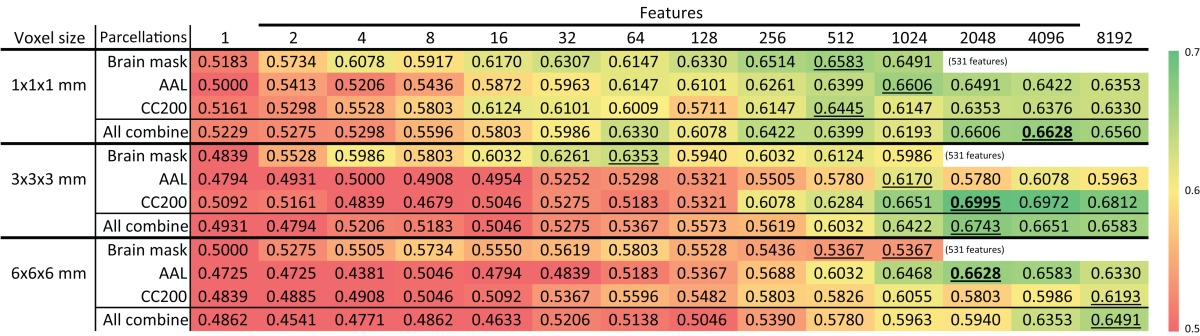

All parcellation (i.e., brain mask, AAL, CC200) results were built by combining LBP-TOP features with R1 + R2 + R3 and the ART non-linear registration method. **All combine** denotes the combination of all features from different parcellations (i.e., brain mask, AAL, and CC200) and various radii. The highest accuracy for each resolution is denoted by the bold number. The highest accuracy for each row is underlined. Other abbreviations are as follows: **AAL**, automated anatomical labeling template; and **CC200**, spatially constrained parcellation based on rs-fMRI.

### Tissue types

To determine the most discriminative tissue type within the brain, models based on GM, WM, and CSF probability maps were tested. These results are shown in Table 7. In most cases, the structural differences found in the GM data provided the highest discriminative power for separating ADHD from TDC subjects. The McNemar's test between different tissue types do not show significant difference while using whole brain and AAL parcellations, but show significant difference in some cases using CC200 parcellations (**Table S6**).

**Table 7:** **The ADHD-TDC classification accuracy of models based on the probability map of different brain tissues using the ART non-linear registration method**.

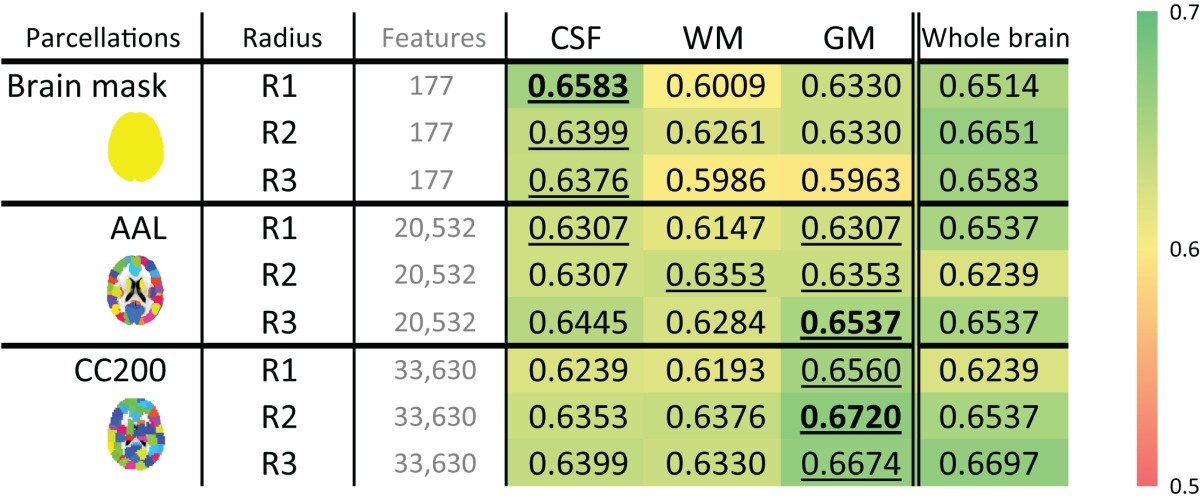

The highest accuracy obtained for each resolution is noted in bold. The highest accuracy for each row is underlined. Abbreviations are as follows: **R1**, **R2**, and **R3**, the LBP-TOP radii in mm; **AAL**, automated anatomical labeling template; and **CC200**, a spatially constrained parcellation based on rs-fMRI.

## Discussion

The prevalence of ADHD around the world is highly heterogeneous. Polanczyk et al. (2007) have shown that this variability may be explained primarily by the use of differing ADHD diagnostic criteria and collateral sources of information. Additionally, geographic location also plays a role in the variability of ADHD prevalence around the world (Faraone et al., 2003; Polanczyk et al., 2007).

Based on the research of Polanczyk et al., estimations of ADHD prevalence rates using the DSM-III-R or ICD-10 criteria are significantly lower than when using other criteria, such as those of the DSM-IV. Additionally, the use of different collateral sources of information, such as parents, teachers, subjects, the best-estimate procedure, the “and rule (parent and teacher),” or the “or rule (parent or teacher),” can also significantly affect the estimate of ADHD (Polanczyk et al., 2007).

The ADHD-200 global competition dataset was pooled from research sites all over the world. The organizers of the competition went to great lengths to maintain the consistency of the dataset. Nevertheless, for various historical reasons, including the use of different benchmarks at each site, it is difficult to use the same procedure to diagnose ADHD around the world (Table 1). However, the worldwide diagnosis of ADHD reflects an objective reality from which ADHD classification models can be built and evaluated.

While constructing classification models based on machine learning approaches, the inconsistency of diagnostic criteria may introduce so-called class label noise, which may seriously diminish accuracy. Class label noise may be the most important contributor to low accuracy in the ADHD-200 Global Competition.

While constructing our ADHD diagnostic tool based on brain images, we found it difficult to compare the rs-fMRI data from different research sites due to differences in image resolution, slice thickness, time points utilized, and image quality. Moreover, the complex preprocessing steps of fMRI data analysis also introduce hardships that can affect the results. Finding the optimal preprocessing strategy to provide the most useful information for building a classifier is a time-consuming process. Therefore, we chose anatomical data rather than rs-fMRI data to mine useful information from brain morphological changes. The resulting classification model based on morphological changes was found to be competitively accurate in discriminating ADHD from TDC subjects. Our results demonstrate that using features based on LBP-TOP data to train the linear SVM can result in greater discriminative power than using features based on rs-fMRI data. The resulting accuracies based on LBP-TOP features are better than those based on rs-fMRI data (Table 2).

### Robust to registration method

The robustness of the registration methods when using LBP-TOP features with ADHD data is notable. Although the model based on the ART non-linear registration method proved to be the most accurate, the models based on linear registrations (FLIRT with 9-DOF and 12-DOF) also performed well in our tests (Table 2). This finding demonstrates the stability of the LBP-TOP to registration methods. Due to the large interindividual variability of the human brain, the registration step of MRI brain data analysis is both critical and challenging (Uylings et al., 2005). Aside from the linear registration method, more than a dozen non-linear registration methods have been developed in recent years, but a perfect registration method does not yet exist (Klein et al., 2009).

However, after performing a perfect registration, no structural differences should exist between subjects. Therefore, a good index for morphological changes should not be based on perfect non-linear registration methods. This property of LBP-TOP might provide a simple and efficient way to compare brain morphology with linearly registered brains.

### Global effects of ADHD?

To introduce different spatial context information, we utilized several parcellation strategies in this study. Unexpectedly, the models using only the distribution of whole-brain features usually demonstrated the highest accuracy in our tests (Tables 2, 3, and 4). Adding parcellation information did not improve the resulting models.

Our results imply that morphological changes in the ADHD brain may affect the whole-brain texture distribution. Further research should be performed to confirm these findings. Theoretically, introducing spatial context information can provide higher accuracy if there are significant structural brain changes in several brain regions. Published structural imaging studies, summarized in two meta-analyses (Valera et al., 2007; Ellison-Wright et al., 2008), have failed to find robust brain changes between ADHD and control subjects. Meta-analyses can help in identifying brain regions that may be the most abnormal in ADHD subjects. However, it is difficult to build a robust discriminative model of ADHD based only on such selected regions.

### Combining models using feature selection

Consider the results of 1 NN and linear-SVM in Table 2. 1 NN uses features as they have same weights, whereas the linear-SVM assigns various weights to them. The results might imply that, with linear registration, use all features with same weight (1 NN) cannot provide good results. However, we can make some features more important to make a better classifier (linear-SVM). Only few features might be needed to build a sufficient good classifier in this problem.

To find the most important features and to improve the robustness and efficiency of our model, we used linear-SVM to rank the overall extracted features, and we made an effort to choose the most important features from which to build a better classification model. Moreover, using the feature selection method, we combined models from different point of view to construct a more general model. The results of our tests show that it is useful to combine features to build better models (Tables 5 and 6). Moreover, we only need few features to build sufficient good classifiers (Table 5). To build a simpler and more robust model, we combined different LBP-TOP features to provide better accuracy. However, when dealing with too many features, the over-fitting effect came into play due to the insufficient number of subjects in this study (436 subjects). In most cases, greater accuracy was not gained by combining more than 4096 features.

### Most discriminative tissue

To determine the most useful brain tissue for discriminating ADHD from TDC subjects, models based on GM, WM, and CSF probability maps were tested. These results are shown in Table 7. In most cases, GM-based structural difference provided the greatest discriminative power.

LBP-TOP extracted morphological data based on the distribution of various curvatures, edges, dots, corners, and the content size of the specific region (Figure 2). Most of this information may come from the complex patterns of cortical folding, which essentially dominates GM morphology. Therefore, we suggest that the primary morphological information utilized by our model may come from gyrification patterns. Wolosin et al. have previously shown different folding indices for ADHD compared with control subjects (Wolosin et al., 2009).

## Conclusions

In this study, we approached the ADHD classification problem by working to find a simple method that could provide sufficient discriminative power. We determined that information derived from texture analysis of brain morphology could be used to distinguish ADHD from TDC subjects. An approach based on structural images is simpler than one based on functional data, and the data are easier to obtain making such an approach potentially more useful in the clinical environment. Our results demonstrate that structural brain data may be another treasure-trove in the ADHD-200 global competition dataset.

Although the accuracy of the models presented in this study are far from being useful clinically, texture difference-based feature extraction may point the way toward a simple and efficient method for determining morphological brain changes. We have demonstrated that LBP-TOP is a good candidate to build a discriminative classification model based on structural brain changes.

### Conflict of interest statement

The authors declare that the research was conducted in the absence of any commercial or financial relationships that could be construed as a potential conflict of interest.

## Figures and Tables

**Table S1:** **Summary of the ADHD and TDC subjects used in this study**. Abbreviations are as follows: **TDC**, typically developing children; **KKI**, the Kennedy Krieger Institute; **NeuroIMAGE**, the NeuroIMAGE sample; **NYU**, the New York University Child Study Center; **OHSU**, Oregon Health and Science University; **Peking_1**, **Peking_2**, and **Peking_3**, Peking University; and **Pittsburgh**, the University of Pittsburgh.

**Table S2:** **The age distribution of subjects used in this study**. **TDC**, typically developing children.

**Table S3:** **List of all subjects**. Abbreviations are as follows: **dx**, diagnosis; **0**, typically developing children (TDC); **1**, ADHD combined-type; **3**, ADHD inattentive-type; **KKI**, the Kennedy Krieger Institute; **NeuroIMAGE**, the NeuroIMAGE sample; **NYU**, the New York University Child Study Center; **OHSU**, Oregon Health and Science University; **Peking_1**, **Peking_2**, and **Peking_3**, Peking University; and **Pittsburgh**, the University of Pittsburgh.

**Table S4:** ***p*-values of McNemar's test of 1NN models and linear-SVM models based on LBP-TOP features between different radii (R1, R2, R3)**. The *p*-values without under lines denote the accuracies of R3 are bigger than R2, the accuracies of R3 are bigger than R1, and the accuracies of R2 are bigger than R2. The underlined *p*-values show the inverse relationship. Bold *p*-values correspond to significant results (*p*-value < 0.05). **DOF9**, and **DOF12**, linear registration with 9, and 12 degree of freedom, respectively; **ART**, non-linear registration performed by Automated Registration Tool; **AAL**, automated anatomical labeling template; and **CC200**, spatially constrained parcellation based on rs-fMRI.

**Table S5:** ***p*-values of McNemar's test of 1NN models and linear-SVM models based on LBP-TOP features between different registrations**. The *p*-values without under lines denote the accuracies of ART are bigger than DOF9, the accuracies of ART are bigger than DOF12, and the accuracies of DOF12 are bigger than DOF9. The underlined *p*-values show the inverse relationship. Bold *p*-values correspond to significant results (*p*-value < 0.05). **DOF9**, and **DOF12**, linear registration with 9, and 12 degree of freedom, respectively; **ART**, non-linear registration performed by Automated Registration Tool; **AAL**, automated anatomical labeling template; and CC200, spatially constrained parcellation based on rs-fMRI.

**Table S6:** ***p*-values of McNemar's test of results in Table 6**. The *p*-values without under lines denote the accuracies of GM are bigger than CSF and the accuracies of GM are bigger than WM. The underlined *p*-values show the inverse relationship. Bold *p*-values correspond to significant results (*p*-value < 0.05). **DOF9**, and **DOF12**, linear registration with 9, and 12 degree of freedom, respectively; **ART**, non-linear registration performed by Automated Registration Tool; **AAL**, automated anatomical labeling template; and CC200, spatially constrained parcellation based on rs-fMRI.
